# Aetiology of genital ulcer disease and associated factors among Mthatha public clinic attendees

**DOI:** 10.4102/sajid.v37i1.444

**Published:** 2022-12-07

**Authors:** Thembisa R. Tshaka, Ravesh Singh, Teke R. Apalata, Zizipho Z.A. Mbulawa

**Affiliations:** 1 Department of Human Biology, Faculty of Health Sciences, Walter Sisulu University, Mthatha, South Africa; 2 Department of Microbiology, National Health Laboratory Services, KwaZulu-Natal Academic Complex, Inkosi Albert Luthuli Central Hospital, Durban, South Africa; 3 School of Laboratory Medicine and Medical Sciences, College of Health Sciences, University of KwaZulu-Natal, Durban, South Africa; 4 National Health Laboratory Service, Nelson Mandela Academic Hospital, Mthatha, South Africa; 5 Department of Laboratory Medicine and Pathology, Faculty of Health Sciences, Walter Sisulu University, Mthatha, South Africa; 6 UCT-MRC Gynaecological Cancer Research Centre, Faculty of Health Science, University of Cape Town, Cape Town, South Africa

**Keywords:** genital ulcer disease, sexually transmitted infections, ulcerating pathogens, human immunodeficiency virus, herpes simplex virus

## Abstract

**Background:**

Genital ulcer disease (GUD) is a sexually transmitted disease characterised by ulcerating lesions. Despite the introduction of sexually transmitted infections (STIs) syndromic management approach into primary healthcare in South Africa (SA) in 1995, the prevalence of STIs in South Africa remains high.

**Objectives:**

The study investigated the aetiology of GUD and factors influencing it among public community health centre (CHC) attendees in the Eastern Cape, South Africa.

**Method:**

A total of 105 participants were recruited among individuals presenting with GUD from three CHCs located in the Eastern Cape Province, South Africa. Blood and genital ulcer samples were collected from consented participants. Blood samples with suitable sera were tested for human immunodeficiency virus (HIV) and syphilis. Herpes simplex virus types 1/2 (HSV–1/2), *Chlamydia trachomatis, Treponema pallidum, Haemophilus ducreyi* and *Klebsiella granulomatis* were detected in nucleic acid extracted from genital ulcer specimens.

**Results:**

Out of the 98 samples with suitable sera, 55.1% and 8.2% were HIV and syphilis seropositive, respectively. Ulcerating STI pathogens were detected in 31.4% of the study participants. Herpes simplex virus type 2 was the most detected pathogen (16.2%) followed by *Chlamydia trachomatis* (10.5%), HSV-1 (8.6%), *Haemophilus ducreyi* (8.6%) and *Treponema pallidum* (6.7%). Multiple pathogens were detected in 13.3% of participants. Detected multiple ulcerating pathogens were common among HIV-positives (*p* = 0.016).

**Conclusion:**

Molecular methods for diagnosing pathogens have the potential to improve the management of GUD. Data generated from this study would contribute to the limited data on GUD in the Eastern Cape Province. Further research with a larger sample size is recommended.

**Contribution:**

Data generated would contribute to the limited data on GUD in the Eastern Cape province, South Africa.

## Background

Genital ulcer disease (GUD) is a sexually transmitted disease (STD) characterised by ulcerating lesions on the genital area, perineum or perianal skin.^1,2^ Genital ulcers may also develop following non-infectious agents, for example, sexual trauma and fixed drug eruptions.^2,3^ When genital ulceration is a result of an infection, the cause can be an STD such as genital herpes caused by herpes simplex virus type 2 (HSV-2), syphilis caused by *Treponema pallidum*, chancroid caused by *Haemophilus ducreyi*, lymphogranuloma venereum (LGV) caused by *Chlamydia trachomatis* serotypes L1-3 and donovanosis caused by *Klebsiella granulomatis*.^4^ Herpes simplex virus type 1 (HSV-1) can also be isolated in genital lesions.^5,6,7^

Sexually transmitted infections (STIs) are considered a burden to the public health sector, especially in low- and middle-income countries. Asymptomatic cases of STI are also prevalent and can be transmitted, and they have a significant negative impact on STI management.^8,9^ Management and treatment of STIs are costly to healthcare services.^8^ In 2018, the World Health Organisation (WHO) estimated one million daily cases of STIs, worldwide.^10^ In rural KwaZulu-Natal, South Africa (SA), a survey conducted among youth revealed a high burden of STIs with *Chlamydia* being the highest.^11^ It was found that syphilis cases among antenatal care attendees were not declining as the cases rose from 1.6% in 2011 to 2.0% in 2015.^12^ There is a reported noticeable decline in the prevalence of chancroid worldwide although the infection might still occur in some African and Caribbean regions.^13,14^ Genital herpes was the relatively prevalent aetiology of genital ulcer syndrome.^15^ Herpes simplex virus type 2 is the major cause of GUD and a highly prevalent STI, worldwide.^16^ In one South African study, 65.2% of GUD cases had ulcer-derived STI pathogens, with HSV accounting for 60.7% of these cases, followed by *Treponema pallidum* (3.9%), *Chlamydia trachomatis* L1-3 (0.9%) and *Haemophilus ducreyi* (0.5%).^17^ The proportion of GUD due to bacterial pathogens had dramatically become less in sub-Saharan Africa.^18,19^

Genital ulceration may be a predisposing or concomitant factor in transmitting HIV.^3,20,21^ With STIs, the risk of male–female HIV transmission increases; moreover, the female–male transmission becomes even higher.^22^ The HIV infection enhancement can be because of various processes, such as the disturbance of normal epithelial barriers with ulcerations, causing the recruitment of HIV-susceptible T-lymphocytes or macrophages to the infected area as part of the host’s immune response.^22,23^ Human immunodeficiency virus-infected persons are more likely to be coinfected with chronic herpesviruses, which replicate periodically producing viable herpes virions.^24^ A South African study revealed that contracting either HIV or HSV-2 will encourage infection by the other.^25^

The GUD epidemiology is also influenced by sexual partners’ gender, socioeconomic factors, multiple/increased sexual companions, status on HIV and local prevalence, drug use, limited prevention, inadequate knowledge of STIs, commercial sex and circumcision.^26^ Young people are at increased risk of acquiring STIs because of their risky behaviour, which is an important health and social concern.^27,28^ High rate of unprotected sex among the heterosexual and homosexual population is driven by several factors, among which there is fear of being considered unfaithful in the relationship and money and gifts in exchange for sex.^29^

The syndromic STI management approach is beneficial, but it limits the opportunities for diagnosing asymptomatic STIs.^9,30^ Syndromic STI management is the diagnosis of STIs based on symptoms and signs, which subsequently leads to their treatment, with point-of-care therapies to treat the majority of microbes that produce specific syndromes without confirmation with the laboratory tests.^31^ It was introduced into South African primary healthcare in 1995; despite this introduction, the burden of STIs remains high. The WHO recommended that syndromic management algorithms be regularly re-evaluated through performing aetiological and antimicrobial resistance periodically.^15^ Acyclovir was added for genital herpes patients with first episodes of the disease^13^; however, treatment failures were reported.^5,6^

There is limited data on GUD in the King Sabata Dalindyebo local municipality (KSDLM), Eastern Cape province of SA, despite the strong evidence that GUD presents a major health challenge. The lack or shortage of evidence-based data in this locality triggered an interest in investigating the aetiology of GUD and its associated factors, and hence this study among public community health centre (CHC) attendees.

## Research methods and design

### Study setting and population

The study was conducted in the KSDLM in the OR Tambo district municipality. Participants were from Gateway, Ngangelizwe and Stanford Terrace CHCs. These CHCs were selected from other KSD CHCs based on the highest number of patients seen between April 2016 and August 2016 presenting with STIs according to the statistics by the KSD health department.

Participants presenting with genital ulcers were recruited between May 2018 and July 2019 during their routine visits at the CHCs. Participants were recruited by educating all CHC attendees using GUD posters. Those who self-reported to have the signs and symptoms of GUD (visible, unhealed ulcers) were physically examined and included in the study. Recruitment and informed consent processes were conducted in English and isiXhosa, a locally spoken language. Trained HIV counsellors conducted the pre- and post-HIV counselling at the CHCs.

Participants were interviewed privately to collect socio-demographic and clinical information, followed by clinical examination and sample collection by a research nurse assisted by the investigator. Structured questionnaire forms were administered, explained and completed with the assistance of the research nurse and/or investigator. The nurse performed the clinical examination. Venous blood was collected into plain tubes. Genital ulcers were swabbed using sterile Dacron swabs (Clinical Sciences Diagnostics Ltd, Booysens, SA) and were put in transport medium, stored at −20 °C and transported to the University of KwaZulu-Natal, Microbiology laboratory, Durban, SA for analysis.

### Screening of HIV and syphilis

Blood specimens were centrifuged and the sera stored at −80 °C prior to testing for syphilis and HIV. Rapid plasma reagin (RPR) test (Fortress Diagnostics Limited, United Kingdom) was used to test for syphilis followed by *Treponema pallidum* haemagglutination assay (TPHA) as a confirmatory test (Fortress Diagnostics Limited, United Kingdom). HIV was tested using Onsite^TM^ HIV 1/2 Ab Plus Combo Rapid Test (CTK Biotech, Inc., Poway, California, United States). The tests were all performed following the manufacturer’s instructions. Rapid plasma reagin /TPHA seropositive participants were referred to the CHC for management.

### DNA extraction

Phenol-chloroform (ThermoFisher Scientific) method was used to extract DNA from the genital ulcer swabs according to the manufacturer’s instructions. Genital ulcer disease swabs were resuspended in 500 μL 20% sodium dodecyl sulphate (SDS) and homogenised (Benchmark Scientifics, Bead Blaster 24 homogeniser, 4 pulses × 30 s; 4 m/s; inter-time 10 s; ambient temperature). Samples were centrifuged at 3000 g for 10 min, and the supernatant (500 μL) was transferred into a clean vial. The homogenate (300 μL) was transferred to a new tube, and 300 μL of UltraPure™ phenol:chloroform:isoamyl alcohol (25:24:1, volume per volume [v/v]) (Invitrogen™ UltraPure™) was added and vortexed vigorously for 10 s, microcentrifuged for 3 min at maximum speed, room temperature. The supernatant (300 μL) was transferred to a new tube containing 100 mM of sodium acetate (Merck), 20 μg of glycogen (Invitrogen) and two volumes of absolute ethanol (Merck). The mixture was incubated in ice for 30 min to precipitate the DNA, after which the samples were centrifuged at 15 900 g for 30 min, and the supernatant was discarded. After the evaporation of the ethanol, the DNA was resuspended with 30 μL of ultrapure sterile water and stored at −70 °C. DNA concentration and quality were determined using a Nanodrop 2000 Spectrophotometer (ThermoScientific, Waltham, Massachusetts, United States).

### Detection of Genital ulcer disease pathogens

The master mix for HSV-2 (assay ID: Vi04646232_s1), *Chlamydia trachomatis* (Ba04646249_s1), HSV1, *Treponema pallidum, Haemophilus ducreyi* and *Klebsiella granulomatis* was prepared by adding 5 μL polymerase chain reaction (PCR)-grade water (Qiagen, Germany), 0.5 μL FAM-labelled probe/primer mix, 2.5 μL Fast Start 4 × probe master mix (ThermoFisher, Part No. 4444434) and 2 μL DNA to make a volume of 10 μL per sample. Amplification was performed at 95 °C for 30 s followed by 45 cycles comprising denaturation at 95 °C for 3 s and annealing at 60 °C for 30 s. Detection of amplified fluorescent products was carried out at the end of the annealing phase. Published primers and probes^43,44^ were used for the detection of HSV-1, *Chlamydia trachomatis, Treponema pallidum, Haemophilus ducreyi* and *Klebsiella granulomatis* (Table 1). Clinical samples that were PCR positive or negative for HSV-1, HSV-2, *Treponema pallidum, Haemophilus ducreyi, Chlamydia trachomatis* or *Klebsiella granulomatis* were included as internal controls. *Klebsiella granulomatis* was detected using conditions as described by Carter and colleagues.^32^

**TABLE 1:** List of primers and probes used to detect ulcerating pathogens in the study.

PCR target	DNA target	Primers and probe	Sequences (5’ to 3’)
HSV1	gB region	HSV1-f	GCAGTTTACGTACAACCACATACAGC
		HSV1-r	AGCTTGCGGGCCTCGTT
		HSV1-p	FAM- CGGCCCAACATATCGTTGACATGGC-MGB
*Treponema pallidum*	GenBank sequence	TP-Zh-131-f	GCCTTTGAGATGGGGATAGC
	(M88726.1)	TP-Zh-245-r	GTCGCAGGCTCATCTCTGA
		TP-Zh-220-p	FAM-CCGCAGCCCCTTTCCTCTCA-MGB
*Haemophilus ducreyi*	GenBank	HD-Zh-992-f	ACATCCATAGAAGAACTCAGAGATGA
	sequences (M75078.1)	HD-Zh-1150-r	TTGAGTTCCCATCAYTACATGCT
		HD-Zh-1022-p	FAM-GTGCCTTCGGGAACTATGTGACAGGT-MGB
*Chlamydia trachomatis*	*Pmp* gene	LGV-f	CTG TGC CAA CCT CAT CAT CAA
		LGV-r	AGA CCC CCT CCG AGC ATC ACT
		LGV-p	FAM-CCT GCT CCA ACA GT-MGB
*Klebsiella granulomatis*	phoE	KG-f	CTA TGA CAG CAA GGA TGG CGA
		KG-r	CAG ACC GAA GTC GAA CTG ATA CTG

*Source:* Adapted from Glatz M, Juricevic N, Altwegg M, et al. A multicenter prospective trial to asses a new real-time polymerase chain reaction for detection of Treponema pallidum, herpes simplex-1/2 and Haemophilus ducreyi in genital, anal and oropharyngeal ulcers. Clin Microbiol Infect. 2014;20(12):O1020-7 and Knauf S, Batamuzi EK, Mlengeya T, et al. Treponema infection associated with genital ulceration in wild baboons. Vet Pathol. 2012;49(2):292–303.

HSV1, Herpes simplex virus type 1; DNA, Deoxyribonucleic acid; LGV, lymphogranuloma venereum; PCR, polymerase chain reaction.

### Data analysis

All variables were captured and coded in Microsoft excel. Single infection was defined as infection with one pathogen type, while multiple infection was defined as two or more pathogen types in the same sample. GraphPad Prism Software v8.0.1.244 statistical software was used to perform chi-squared for trends, Fisher’s exact to compare the proportion between variables and the relative risk (RR). A *p* value ≤ 0.05 was used to indicate statistical significance.

### Ethical considerations

All study aspects were approved by the Human Research Ethics Committee of Walter Sisulu University (HREC: 015/2016), EC Department of Health (EC_2016RP26_934) and KSD Department of health sub-district, Mthatha. Written informed consent was obtained from participants.

## Results

### Demographic characteristics of the population

A total of 105 public CHC attendees with GUD participated in the study. The majority were from Gateway CHC (91.4%, 96/105), with only 6.7% (7/105) from Ngangelizwe CHC and 1.9% (2/105) from Stanford Terrace CHC. Participants’ age ranged from 16 to 57 years, with a median of 28 years. Most participants were single (79%, 83/105), female (74.3%, 78/105), living in an informal settlement (54.3%, 57/105) and rural area residents (26.7%, 28/105). The education level ranged from primary to tertiary, with the highest number at secondary level (50.5%, 53/105). The bulk of the participants were unemployed (38.1%, 40/105) and students (26.7%, 28/105). The majority of the participants reported not being substance abusers (alcohol, drugs, smoking of any kind, 68.6%, 72/105), followed by those drinking alcohol with/without dagga (21.9%, 23/105). The majority of the participants were seen to be engaging in unprotected sex (87.6%, 92/105). Almost half the population (47.6%, 50/105) had two or more sexual partners at a time (Table 2).

**TABLE 2:** Demographic characteristics of the study population.

Variable	*n*	%
**Gender**		
Female	78	74.3
Male	27	25.7
**Age**		
16–25 years	41	39.0
26–35 years	41	39.0
36–57 years	23	21.9
**Partners age range**		
< 18–39 years	80	76.2
> 40 years	15	14.3
Missing data	10	9.5
**Marital status**		
Single	83	79.0
Married/widowed/divorced	17	16.2
Missing data	5	4.8
**Residence**		
Informal settlement	57	54.3
Rural	28	26.7
Semi-urban	9	8.6
Urban	11	10.5
**Education**		
Primary	7	6.7
Secondary	53	50.5
Matric	21	20.0
Tertiary	22	21.0
Missing data	2	1.9
**Occupation**		
Employed	35	33.3
Unemployed	40	38.1
Student	28	26.7
Missing data	2	1.9
**Substance abuse**		
Alcohol, dagga, cigarette	28	26.7
Clean	72	68.6
Missing data	5	4.8
**Protected sex**		
Yes	7	6.7
No	92	87.6
Missing data	6	5.7
**No. of sexual partners**		
1	49	46.7
≥ 2	50	47.6
Missing data	6	5.7

### Prevalence of laboratory detected ulcerating sexually transmitted infection pathogens and factors among public community health centre attendees with genital ulcer disease

Out of the 98 sera, *Treponema pallidum* was detected in 33 (8.2%) samples, while 33 of the 105 ulcer swabs (31.4%) were positive for ulcerating STIs. Multiple pathogens were detected in 13.3% (14/105) of specimens. The most commonly detected ulcerating STI was HSV-2, 16.2%, (17/105) followed by *Chlamydia trachomatis* L1-3, 10.5% (11/105), HSV-1, 8.6% (9/105), *Haemophilus ducreyi*, 8.6% (9/105) and *Treponema pallidum*, 6.7% (7/105) and no *Klebsiella granulomatis* was detected. Prevalence of ulcerating STIs was higher among GUD HIV-negative compared to HIV-positive participants (40.9%, 18/44 vs 22.2%, 12/54, *p* < 0.001). The ulcerating STIs were detected as single infections among GUD HIV-negative (*p* < 0.001) and as multiple infections among the HIV-positive (*p* = 0.016) population (Table 3).

**TABLE 3:** Prevalence of ulcerating pathogens among public community health centre attendees with Genital ulcer disease.

Ulcerating pathogens	Overall (*n* = 105)		HIV-negative (*n* = 44)		HIV-positive (*n* = 54)		*p* *
	*n*	%	*n*	%	*n*	%	
Prevalence of GUD pathogens	33	31.4	18	40.9	12	22.2	0.051
Single organisms	19	18.1	18	40.9	5	9.3	**< 0.001**
Multiple organism (2–4)	14	13.3	0	0.0	7	13.0	**0.016**
Herpes simplex virus type 2	17	16.2	9	20.5	7	13.0	0.412
Herpes simplex virus type 1	9	8.6	3	6.8	5	9.3	0.727
*Chlamydia trachomatis* L1-3	11	10.5	4	9.1	6	11.1	> 0.999
*Treponema pallidum*	7	6.7	5	11.4	1	1.9	0.087
*Haemophilus ducreyi*	9	8.6	3	6.8	5	9.3	0.727
*Klebsiella granulomatis*	0	0.0	0	0.0	0	0.0	
Total GUD pathogens detected	53	50.6	24	54.5	24	44.4	0.417

GUD, genital ulcer disease.

* , Compared HIV-positive with HIV-negative. The *p*-values in bold are significant *p* < 0.05.

Herpes simplex virus type 2 was more prevalent among HIV-positive participants than HIV-negatives (13%, 7/54 vs 20.5%, 9/44), and this was not statistically significant, *p* = 0.412. There was no difference among the HIV-positive and HIV-negative participants for HSV-1 (9.3%, 5/54 vs 6.8%, 3/44, *p* = 0.727) and *Haemophilus ducreyi* (9.3%, 5/54 vs 6.8%, 3/44, *p* = 0.727). There was a noticeable difference in the prevalence of *Treponema pallidum* between HIV-positive and HIV-negative participants (1.9%, 1/54 vs 11.4%, 5/44), respectively, and this was not statistically significant, *p* = 0.087 (Table 3).

The prevalence of GUD pathogens was higher among male participants than female participants (48.1% vs 25.6%, RR: 0.75, 95% CI: 0.53–0.98, *p* = 0.053). It was also higher among participants with partners aged < 18–39 years compared with > 40 years (33.8% vs 13.3%, RR: 2.53, 95% CI: 0.84–9.29, *p* = 0.138) and among participants with matric (28.6%, RR: 0.50, 95% CI: 0.08–2.32, *p* = 0.639) or tertiary education level (27.3%, RR: 0.52, 95% CI: 0.09–2.44, *p* = 0.646); however, the associations were not statistically significant (Table 4).

**TABLE 4:** Demographic factors associated with laboratory detected ulcerating Sexually transmitted infection pathogens.

Variable	*N*	GUD pathogens				
		*n*	%	RR	95% CI	*p*
**Gender**						
Female	78	20	25.6			ref
Male	27	13	48.1	0.75	0.53–0.98	0.053
**Age**						
16–25 years	41	13	31.7			ref
26–35 years	41	12	29.3	1.08	0.57–2.08	> 0.999
36–57 years	23	8	34.8	0.91	0.46–1.90	> 0.999
**Partners age range**						
< 18–39 years	80	27	33.8			ref
> 40 years	15	2	13.3	2.53	0.84–9.29	0.138
*Missing data*	*10*	*4*	*40.0*	*0.84*	*0.44–2.10*	*0.732*
**Marital status**						
Single	83	25	30.1			ref
Married/widowed/divorced	17	6	35.3	0.85	0.45–1.85	0.775
*Missing data*	*5*	*2*	*40.0*	*0.75*	*0.34–2.65*	*0.643*
**Residence**						
Informal settlement	57	15	26.3			ref
Rural	28	11	39.3	0.67	0.36–1.28	0.316
Semi-urban	9	4	44.4	0.59	0.29–1.52	0.267
Urban	11	3	27.3	0.96	0.39–2.90	> 0.999
**Education**						
Primary	7	1	14.3			ref
Secondary	53	18	34.0	0.42	0.07–1.66	0.414
Matric	21	6	28.6	0.50	0.08–2.32	0.639
Tertiary	22	6	27.3	0.52	0.09–2.44	0.646
*Missing data*	*2*	*2*	*100.0*	*0.14*	*0.03–0.78*	*0.083*
**Occupation**						
Employed	35	8	22.9			ref
Unemployed	40	16	40.0	0.57	0.28–1.13	0.140
Student	28	8	28.6	0.80	0.35–1.84	0.772
*Missing data*	*2*	*1*	*50.0*	*0.46*	*0.17–2.59*	*0.432*
**Substance abuse**						
Alcohol, dagga, cigarette	28	8	28.6			ref
Clean	72	22	30.6	0.94	0.46–1.76	> 0.999
*Missing data*	*5*	*3*	*60.0*	*0.48*	*0.21–1.40*	*0.304*
**Protected sex**						
Yes	7	1	14.3			ref
No	92	27	29.3	0.49	0.09–1.87	0.669
*Missing data*	*6*	*5*	*83.3*	*0.17*	*0.03–0.73*	*0.029*
**Number of sexual partners**						
1	49	15	30.6			Ref
≥ 2	50	17	34.0	0.90	0.51–1.59	0.831
*Missing data*	*6*	*1*	*16.6*	*1.84*	*0.48–10.52*	*0.660*

Note: Missing values reflect in italics

STI, Sexually transmitted infections; GUD, Genital ulcer disease; RR, relative risk.

Condom use was associated with decreased prevalence of GUD pathogens (14.3% vs 29.3%; RR: 0.49, 95% CI: 0.09–1.87), but this was not statistically significant (*p* = 0.669). The prevalence of GUD pathogens was not influenced by age, residential area, marital status, number of sexual partners or substance abuse (Table 4).

### Factors associated with genital ulcer disease among HIV-positive and HIV-negative participants

**FIGURE 1:**
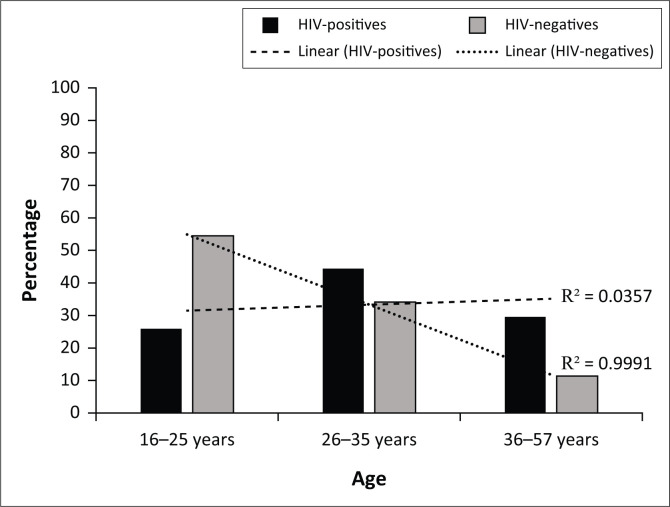
Human immunodeficiency virus status according to age groups among women and men with genital ulcer disease.

Among the GUD participants, 55.1% (54/98) were HIV-positive versus 44.9% (44/98) who were HIV-negative, and this was not statistically significant, *p* = 0.198. A significantly higher population was females in both HIV-positives (77.8%, 42/54 vs 22.2%, 12/54, *p* < 0.001) and HIV-negatives (65.9%, 29/44; 34.1%, 15/44, *p* = 0.005). Interestingly, among female participants, a significantly higher proportion of the population was HIV-positive (59.2%, 42/71) than HIV-negative (40.8%, 29/71, *p* = 0.044); while this was not observed among male participants (*p* = 0.587, Table 5). Among HIV-negatives, the proportion of the GUD population decreased with increasing age significantly (*p* < 0.001) but not among HIV-positives (*p* = 0.683, Figure 1). The majority of the participants were 16–25 years and were HIV-negative (63.2%, 24/38) than HIV-positive (36.8%, 14/38, *p* = 0.038). In contrast, among the 36–57 years’ group, the majority were HIV-positive (76.2%, 16/21) than HIV-negative (23.8%, 5/21, *p* = 0.002, Table 5).

**TABLE 5:** Factors associated with Genital ulcer disease among HIV-positive and HIV-negative participants.

Variable	*N*	HIV-positive			HIV-negative			*p* *
		*n*	%	*p*	*n*	%	*p*	
**Overall**	98	54	55.1		44	44.9		0.198
**Gender**								
Female	71	42	77.8	ref	29	65.9	ref	**0.044**
Male	27	12	22.2	**< 0.001**	15	34.1	**0.005**	0.587
**Age**								
16–25 years	38	14	25.9	0.683	24	54.5	**< 0.001**	**0.038**
26–35 years	39	24	44.4		15	34.1		0.069
36–57 years	21	16	29.6		5	11.4		**0.002**
**Partners age range**								
< 18–39 years	77	39	72.2	ref	38	86.4	ref	> 0.999
> 40 years	14	12	22.2	**< 0.001**	2	4.5	**< 0.001**	**< 0.001**
Missing data	7	3	5.6	**< 0.001**	4	9.1	**< 0.001**	> 0.999
**Marital status**								
Single	77	41	75.9	ref	36	81.8	ref	0.519
Married/widowed/divorced	16	11	20.4	**< 0.001**	5	11.4	**< 0.001**	0.076
Missing data	5	2	3.7	**< 0.001**	3	6.8	**< 0.001**	> 0.999
**Residence**								
Informal settlement	53	28	51.9	ref	25	56.8	ref	0.698
Rural	25	18	33.3	0.079	7	15.9	**< 0.001**	**0.004**
Semi-urban	9	4	7.4	**< 0.001**	5	11.4	**< 0.001**	> 0.999
Urban	11	4	7.4	**< 0.001**	7	15.9	**< 0.001**	0.395
**Education**								
Primary	7	5	9.3	ref	2	4.5	ref	0.286
Secondary	48	28	51.9	**< 0.001**	20	45.5	**< 0.001**	0.153
Matric	21	14	25.9	**0.041**	7	15.9	0.157	0.063
Tertiary	21	7	13.0	0.761	14	31.8	**0.002**	0.063
Missing data	1	0	0.0		1	2.3	> 0.999	
**Occupation**								
Employed	34	25	46.3	ref	9	20.5	ref	**< 0.001**
Unemployed	38	23	42.6	0.847	15	34.1	0.231	0.108
Student	24	5	9.3	**< 0.001**	19	43.2	**0.038**	**< 0.001**
Missing data	2	1	1.9	**< 0.001**	1	2.3	**0.015**	> 0.999
**Substance abuse**								
Alcohol, dagga	21	12	22.2	ref	9	20.5	ref	0.538
Cigarette only	5	4	7.4	0.055	1	2.3	**0.015**	0.206
Clean	68	36	66.7	**< 0.001**	32	72.7	**< 0.001**	0.607
Missing data	4	2	3.7	**0.008**	2	4.5	**0.049**	> 0.999
**Protected sex**								
Yes	5	2	3.7	ref	3	6.8	ref	> 0.999
No	88	49	90.7	**< 0.001**	39	88.6	**< 0.001**	0.175
Missing data	5	3	5.6	> 0.999	2	4.5	> 0.999	> 0.999
**No. of sexual partners**								
1	47	28	51.9	ref	19	43.2	ref	0.098
≥ 2	45	22	40.7	0.335	23	52.3	0.522	> 0.999
Missing data	6	4	7.4	**< 0.001**	2	4.5	**< 0.001**	0.567

* , compares HIV-positives and HIV-negatives. The *p*-values in bold are significant < 0.05.

A high proportion of GUD was seen in the participants with partners aged < 18–39 years compared with > 40 years in both HIV-positives (72.2%, 39/54; 22.2% 12/54, *p* < 0.001) and HIV-negatives (86.4%, 38/44; 4.5%, 2/44, *p* < 0.001). A significant proportion of the GUD students was HIV-negative compared with the HIV-positive (79.2%, 19/24 vs 28.2%, 5/24, *p* < 0.001). While in the employed population with GUD, a high proportion was HIV-positive (73.5%, 25/34 vs 26.5%, 9/34, *p* < 0.001, Table 5). The majority of the population reported single marital status compared with other married/widowed/divorced marital status, in both HIV-positive (75.9%, 41/54; 20.4%, 11/54, respectively, *p* < 0.001) and HIV-negative (81.8%, 36/44; 11.4%, 5/44, respectively, *p* < 0.001) groups. Importantly, among the groups with single marital status, the proportion did not differ when stratified according to HIV status (*p* = 0.519). Similar findings were observed among the married/widowed/divorced marital status group (*p* = 0.076, Table 5).

Among the GUD HIV-positive group, a higher proportion resided in informal settlement compared with the rural; however, this was not statistically significant (51.9%, 28/54; 33.3%, 18/54; *p* = 0.079), while among the HIV-negative population, there was a significantly higher proportion (56.8%, 25/44 vs 15.9%, 7/44; *p* < 0.001). Furthermore, there was a significant higher GUD HIV-positives residing in informal settlement than semi-urban (51.9%, 28/54 vs 7.4%, 4/54; *p* < 0.001) or urban residents (51.9%, 28/54 vs 7.4%, 4/54; *p* < 0.001). Similar findings were observed among the GUD HIV-negatives (56.8%, 25/44; 11.4%, 5/44, *p* < 0.001; 56.8%, 25/44; 15.9%, 7/44; *p* < 0.001), respectively. The rural GUD population showed a significantly lesser proportion of HIV-negatives compared with HIV-positives (28.0%, 7/25; 72.0%, 18/25; *p* = 0.004, Table 5).

Few GUD HIV-positive participants had primary than secondary education (9.3%, 5/54; 51.9%, 28/54; *p* < 0.001) and matric (9.3%, 5/54; 25.9% 14/54, *p* = 0.041) but not tertiary education (9.3%, 5/54; 13.0% 7/54, *p* = 0.761). Among GUD HIV-negative participants, a smaller proportion had primary than secondary education (4.5%, 2/44; 45.5%, 20/44; *p* < 0.001) and tertiary (4.5%, 2/44; 31.8% 14/44, *p* = 0.002) and matric education (4.5%, 2/44; 15.9% 7/44, *p* = 0.063). The GUD HIV-positive employed population had higher prevalence of students (46.3%, 25/54; 9.3%, 5/54; *p* < 0.001) and unemployed population (46.3%, 25/54; 42.6%, 23/54; *p* = 0.847). In contrast, the GUD HIV-negatives had a lower employed population than students (20.5%, 9/44; 43.2%, 19/44; *p* = 0.038) followed by unemployed (20.5%, 9/44; 34.1%, 15/44; *p* = 0.231) but not statistically significant.

Among the GUD HIV-positive population, those that reported clean of substance abuse were significantly higher than alcohol/dagga users (66.7%, 36/54; high 22.2%, 12/54; *p* < 0.001) as also seen in the HIV-negative group (72.7%, 32/44 vs 20.5%, 9/44; *p* < 0.001). A significantly high proportion of GUD was seen in the HIV-positive and HIV-negative population, and they reported unprotected sexual intercourse (90.7%, 49/54 vs 3.7%, 2/54; *p* < 0.001 and 88.6%, 39/44; 6.8%, 3/44; *p* < 0.001, respectively). There was no difference in the number of sexual partners among the GUD HIV-positive and HIV-negative populations (Table 5).

## Discussion

The high number of GUD infections observed among the female participants in the present study could be because of the increased risk of GUD that is associated with the more fragile surface of female reproductive organs.^33^ The observed low proportion of GUD infections among male participants could be because of less willingness to be involved in a research study or few men with GUD attending the CHC. However, it has been reported that among men, clinically GUD diagnosis is a predictor of re-visiting primary prevention facilities.^34^

A third of the GUD population had ulcerating STIs. Studies revealed that the development of genital ulcers might not only be because of STIs but also non-infectious agents.^2,3^ The observed low prevalence of ulcerating pathogens might be because of non-infectious agents^2,3^ or low viral load of the pathogen that resulted in false-negative results.^35,36^ There was more than one type of ulcerating STIs in some specimens, and this was prevalent among HIV-population. Previous studies reported that there could be more than one aetiological agent in any genital/anal/perianal ulcer.^28^ Literature has documented that HIV-population is at increased risk of multiple STIs as pathogens share transmission routes.^37^

Herpes simplex virus type 2 was reported as the most causative organism of GUD. Similar observations were observed in this study. A South African study also indicated that HSV-2 remained the leading cause of ulcerating STI pathogen, supporting the use of acyclovir in the STI syndromic management.^17^ Herpes simplex virus type 1 was also one of the causes of GUD in this study. Studies previously conducted have revealed that HSV-1 can also be isolated in genital lesions,^5,6,7,26^ and this could be because of the increased practice of orogenital sex.^38^ This study revealed that *Chlamydia trachomatis* L1-3 was still a burden in SA. Previous studies have reported *Chlamydia* to be among pathogens causing a high burden of STIs among youth in SA.^11^ *Klebsiella granulomatis* was not detected in this study, a finding similar to a previous study that was conducted among the South African population that also reported negative *Klebsiella granulomatis* in genital ulcer specimens.^39^ The negative results are probably because of public recognition of Donovanosis as a health problem with control measures or as a result of the improvement of health services or living standards.^14^

*Haemophilus ducreyi* was the third-highest ulcerating pathogen in this study. Studies conducted worldwide have reported a decline of chancroid; however, infections might still occur in some African and Caribbean regions.^13,14^ This calls for review or monitoring of the STI syndromic management approach because *Haemophilus ducreyi* still seems to be burdensome. The high prevalence of ulcerating STIs among participants living in a semi-urban and rural area compared with participants in informal settlement and urban area residents has been observed in a study conducted in three clinical research sites in Durban, SA, where HSV-2 prevalence was also high among participants in rural/semi-rural areas of Durban. The higher prevalence in rural areas could be attributed to women who have less access to jobs and may engage in more transactional sex. Furthermore, rural areas have less access to healthcare, including STI treatment and free condoms.^40^ Lower social status, lower education levels and lower income have been associated with increased risk of STIs.^41^ The informal settlement population was more affected by the GUD and with a higher HIV-positivity rate compared with other population groups in this study; however, the prevalence of GUD pathogen was low.

The GUD prevalence was less dominant among participants with primary education, which is contrary to findings from other studies that reported lower education level as a risk factor for STIs.^40^ Similarly, others have reported drug use as one of the factors influencing the epidemiology of GUD,^26^ which is contrary to the current study’s findings, which found no difference in the prevalence of ulcerating STIs among alcohol/dagga/cigarettes and those not using them. Supposedly, this might be because of false reporting. However, the HIV-positivity rate was a bit higher among those taking alcohol/drugs/cigarettes compared to the HIV-negativity rate. Factors such as socioeconomic factors, lack of adequate knowledge of STIs and multiple or increased sexual partners are reported to influence the epidemiology of GUD.^26^ This is supported by high GUD or ulcerating STIs among the unemployed and no-condom use population in this study.

The HIV seroprevalence rate among GUD participants was high (55.1%). The relationship between genital ulcers and HIV acquisition is well documented in the literature.^3,20,21^ Disruption of normal epithelial barriers with ulcerations causes recruitment of HIV-susceptible T-lymphocytes or macrophages to the infected area as part of the host’s immune response.^22^ South African researchers also found the prevalence of HIV co-infection among GUD patients to be high.^17^

Syphilis serology had different results from the nucleic acid detection method. There was not much difference in percentages, but two samples were positive in both serological and nucleic acid tests. The reason could be that syphilis is a multistage STD. The primary stage is characterised by a chancre, where the test’s sensitivity is high in collected nucleic acid test samples. Serological tests might be negative at the primary stage because of the window period between transmission and seroconversion, and as the stages progress, the serology test would be positive.^42^

### Limitations of the study

The small sample size in this study could be a limitation. Therefore, a larger sample size is recommended. The distribution of participants from the three recruitment sites was not equal, and there were few male participants. Therefore, the results could not be generalised to represent the three CHCs. Despite these limitations, this study remains valuable for the EC population. Further research with larger sample size is recommended.

## Conclusion

Herpes simplex virus type 2 was the leading cause of GUD in KSDLM followed by *Chlamydia trachomatis* L1-3, HSV-1 and *Haemophilus ducreyi* and *Treponema pallidum*. The use of molecular methods in diagnosing ulcerating STIs can potentially improve the management of GUD. Sexually transmitted infections/STD and sexual behaviour education interventions remain essential to improve sexual behaviour and reduce the STD burden.
